# Supplementary material for: Phosphatase activity is dispensable for PRL-3-mediated oncogenesis and tumor progression
Source: bioRxiv. 2025 May 18:2025.05.14.654016. Preprint. [Version 1] doi: 10.1101/2025.05.14.654016 (PMC12132575; doi:10.1101/2025.05.14.654016)
Supplement: 1 [file NIHPP2025.05.14.654016V1-supplement-1.pdf]

bioRxiv preprint doi: https://doi.org/10.1101/2025.05.14.654016; this version posted May 18, 2025. The copyright holder for this preprint (which was not certified by peer review) is the author/funder, who has granted bioRxiv a license to display the preprint in perpetuity. It is made available under aCC-BY 4.0 International license.

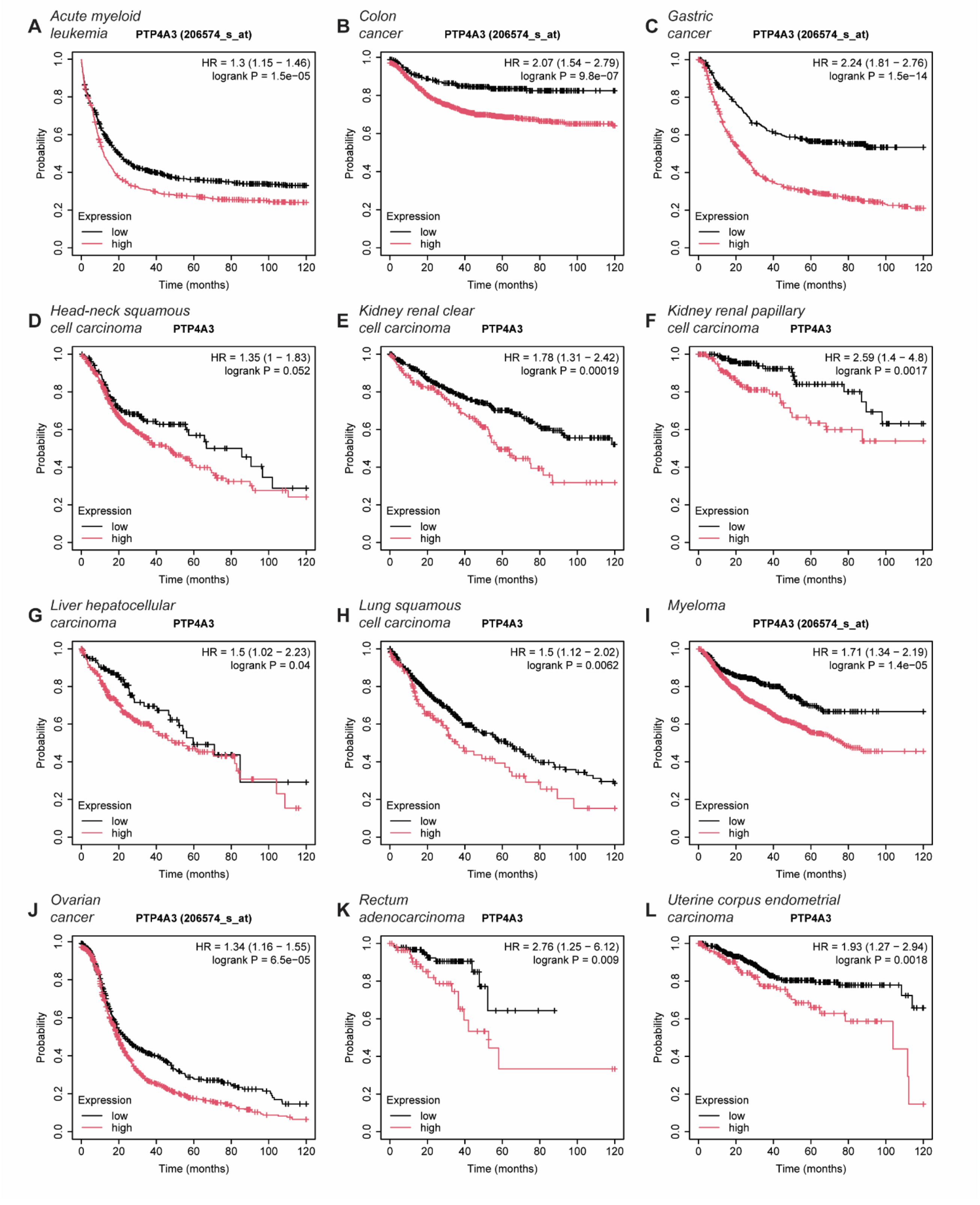

Supplemental Figure 1. High PRL-3/PTP4A3 expression correlates with a poor patient prognosis.
Kaplan-Meier analysis of overall survival in **(A)** acute myeloid leukemia**, (B)** colon cancer, **(C)** gastric cancer, **(D)** head-neck squamous cell carcinoma, **(E)** kidney renal clear cell carcinoma, **(F)** kidney renal papillary cell carcinoma, **(G)** liver hepatocellular carcinoma, **(H)** lung squamous cell carcinoma, **(I)** myeloma, **(J)** ovarian cancer, **(K)** rectum adenocarcinoma, and **(L)** uterine corpus endometrial carcinoma. Sample cohorts are split into PTP4A3 low (black) and high (red) by the median. Hazard ratios were calculated for the PTP4A3 high groups. Data shown is derived from the online tool KM-plotter and pools from microarray (PTP4A3 [206574_s_at]) and RNAseq (PTP4A3) data sets.

bioRxiv preprint doi: https://doi.org/10.1101/2025.05.14.654016; this version posted May 18, 2025. The copyright holder for this preprint (which was not certified by peer review) is the author/funder, who has granted bioRxiv a license to display the preprint in perpetuity. It is made available under aCC-BY 4.0 International license.

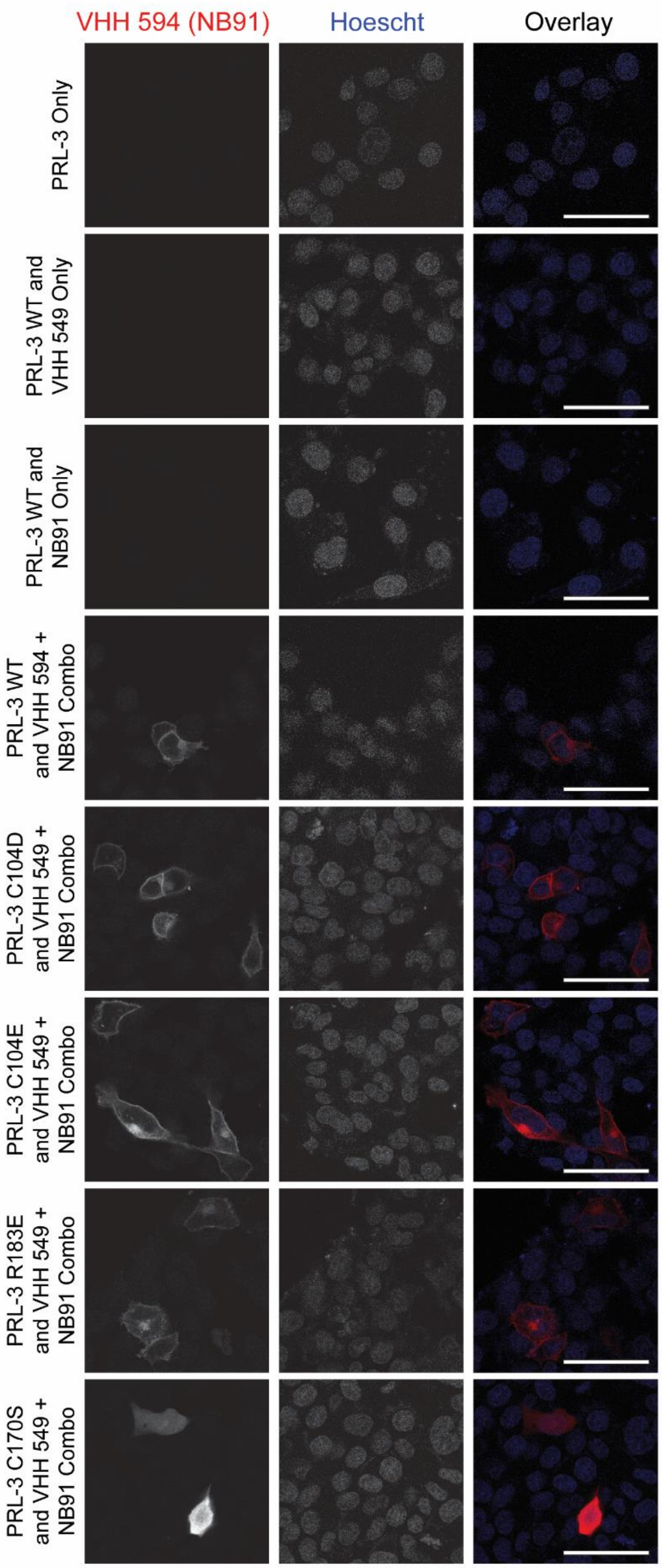

Supplemental Figure 2. PRL-3 point mutations retain the localization patterns of the wild-type protein. Immunofluorescence (IF) images of HCT116 cells transfected with PRL-3 WT or mutants to assess subcellular localization. Nuclei were visualized with Hoechst 33342 (blue). PRL-3 was detected using a PRL-3-specific nanobody (NB91) and visualized with an anti-alpaca AlexaFluor 594 (VHH 594, red). Scale bar, 20 μm.

bioRxiv preprint doi: https://doi.org/10.1101/2025.05.14.654016; this version posted May 18, 2025. The copyright holder for this preprint (which was not certified by peer review) is the author/funder, who has granted bioRxiv a license to display the preprint in perpetuity. It is made available under aCC-BY 4.0 International license.

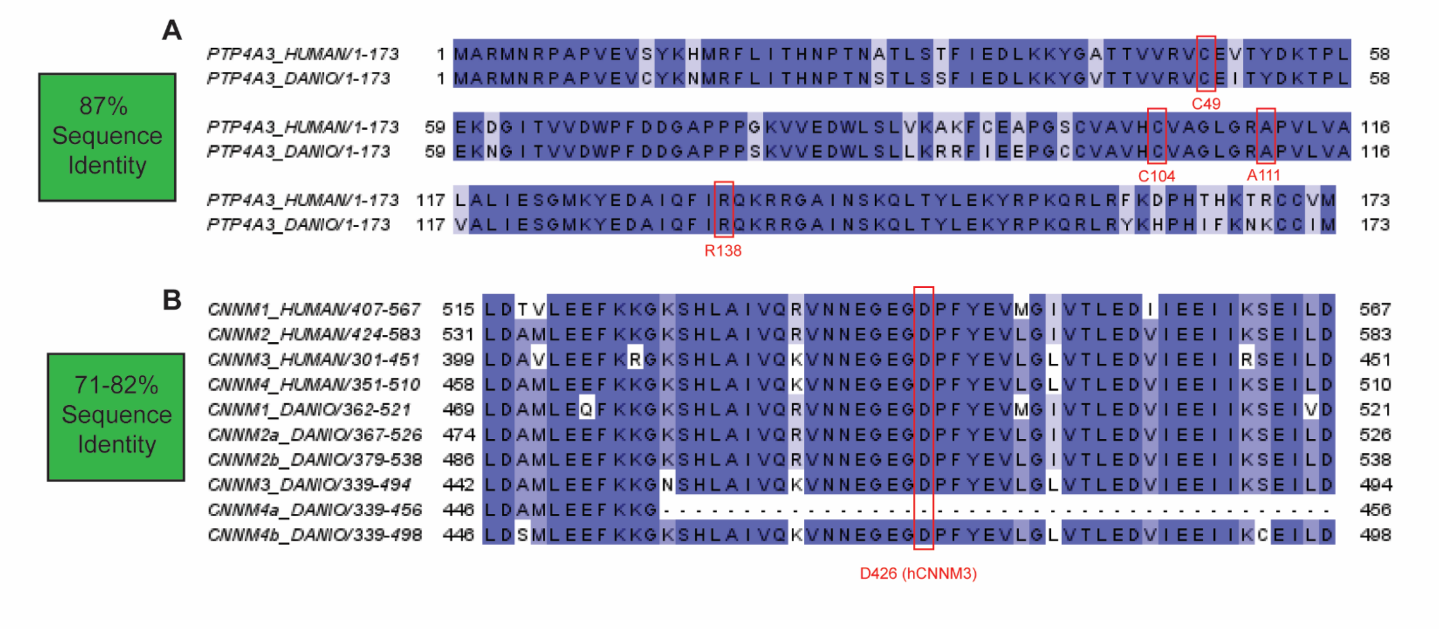

Supplemental Figure 3. Sequence alignments show that PRL-3 and the CBS domain of CNNMs are highly conserved across humans and zebrafish.
(A) Protein sequence alignment of human and zebrafish (*Danio rerio*) PRL-3 (PTP4A3) with residues of interest in red, including the C104 catalytic site and R138, which is critical for CBS-domain binding. **(B)** Protein sequence alignment of the CBS domains of human and zebrafish CNNM proteins. Mutation of D426, shown in red, has been shown to reduce PRL-3 binding.

bioRxiv preprint doi: https://doi.org/10.1101/2025.05.14.654016; this version posted May 18, 2025. The copyright holder for this preprint (which was not certified by peer review) is the author/funder, who has granted bioRxiv a license to display the preprint in perpetuity. It is made available under aCC-BY 4.0 International license.

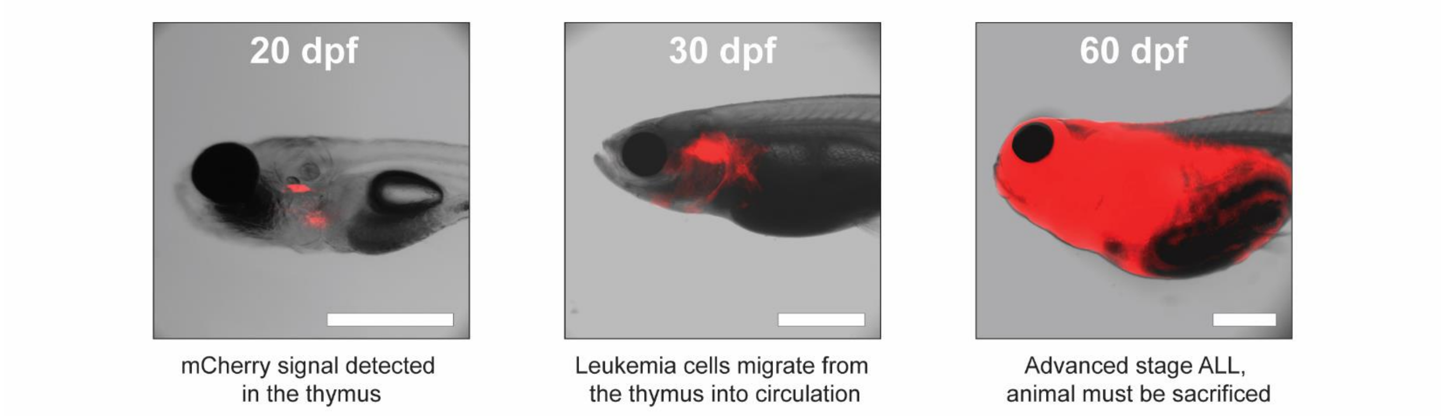

Supplemental Figure 4. Leukemia in the zebrafish T-ALL model initiates in the thymus and disseminates systemically.
Representative images of a single rag2:myc;rag2:mCherry transgenic zebrafish were captured at 20, 30, and 60 days post-fertilization (dpf) to monitor leukemia progression over time. mCherry-positive leukemia cells first accumulate in the thymus and later spread into the circulation. Scale bar, ~1 mm.

bioRxiv preprint doi: https://doi.org/10.1101/2025.05.14.654016; this version posted May 18, 2025. The copyright holder for this preprint (which was not certified by peer review) is the author/funder, who has granted bioRxiv a license to display the preprint in perpetuity. It is made available under aCC-BY 4.0 International license.

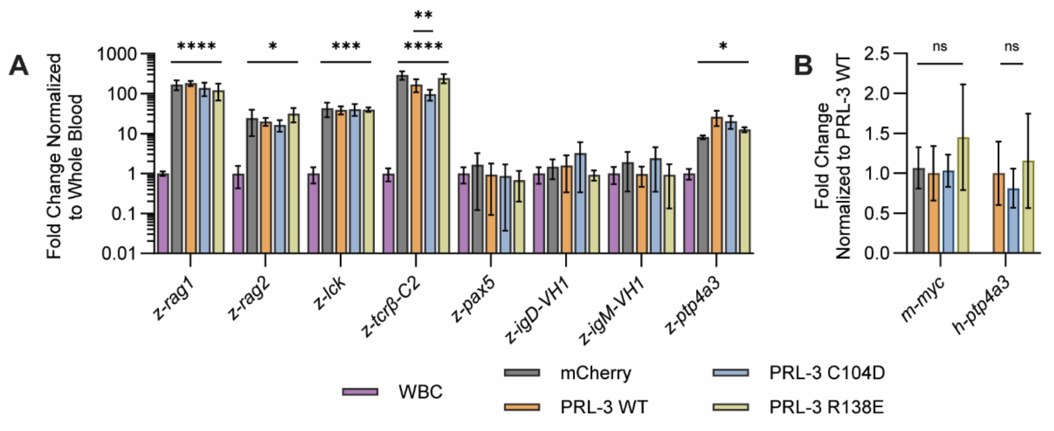

Supplemental Figure 5. The overexpression of PRL-3 mutants has a minimal effect on ALL gene signatures. **(A)** RT-qPCR analysis of endogenous genes involved in lymphoid, T cell, or B cell specification in the indicated ALL samples, normalized to expression in non-cancerous whole blood (WBC) **(B)** RT-qPCR analysis results for injected transgenes normalized to the PRL-3 WT group. Each analysis was performed with samples extracted from six animals per condition. Error bars represent standard deviation. Statistical significance was calculated using GraphPad Prism with an ordinary one-way ANOVA and Tukey's multiple comparisons test. *, p<0.05; **, p<0.01; ***, p<0.001; ****, p<0.0001.

bioRxiv preprint doi: https://doi.org/10.1101/2025.05.14.654016; this version posted May 18, 2025. The copyright holder for this preprint (which was not certified by peer review) is the author/funder, who has granted bioRxiv a license to display the preprint in perpetuity. It is made available under aCC-BY 4.0 International license.

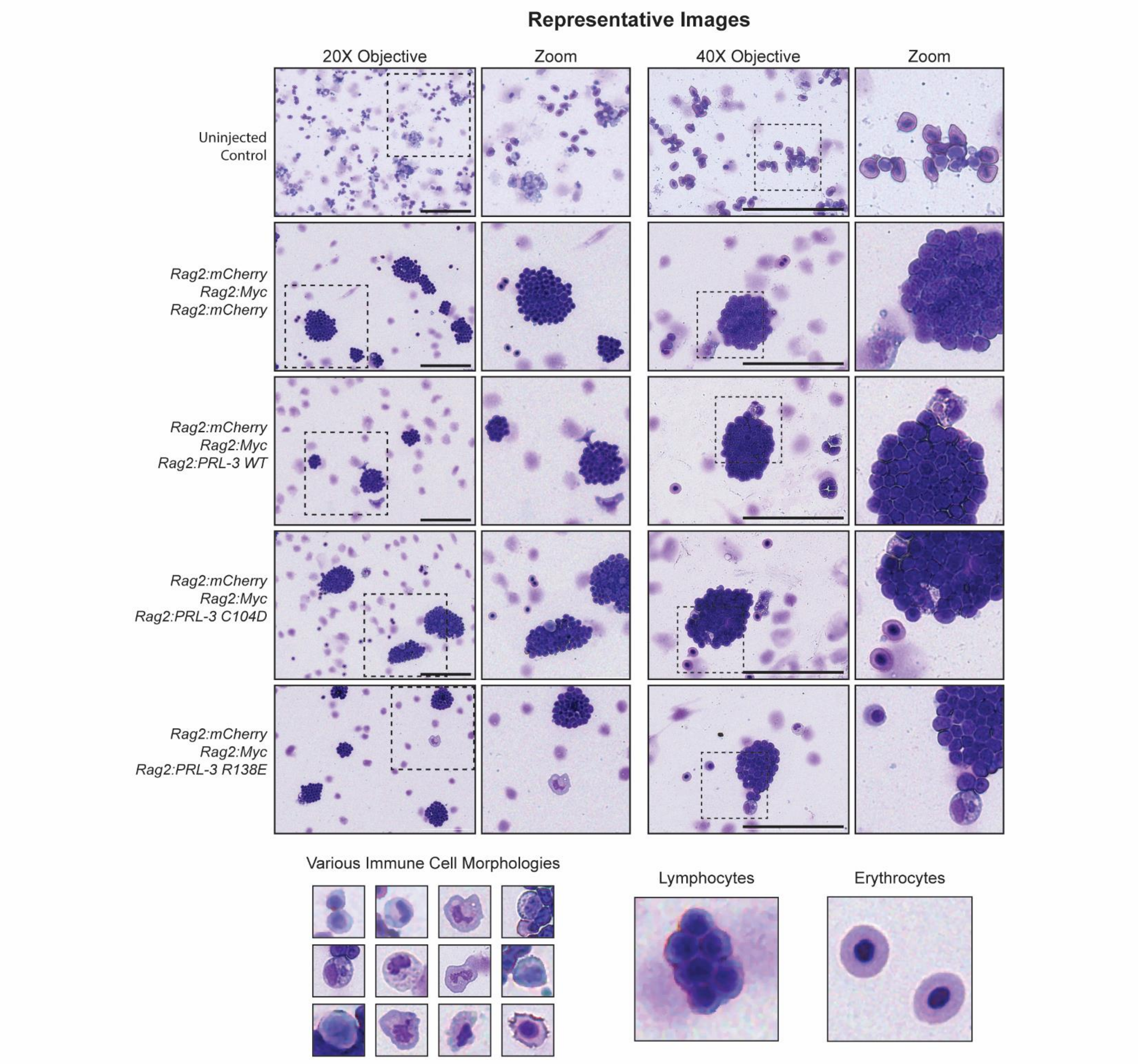

Supplemental Figure 6. Representative May-Grünwald-Giemsa staining of zebrafish ALL samples. Zebrafish acute lymphoblastic leukemia (ALL) samples from each experimental group were stained with May-Grünwald-Giemsa to assess cellular morphology. Images shown are representative of at least two animals per group. Scale bar, 100 μm.

bioRxiv preprint doi: https://doi.org/10.1101/2025.05.14.654016; this version posted May 18, 2025. The copyright holder for this preprint (which was not certified by peer review) is the author/funder, who has granted bioRxiv a license to display the preprint in perpetuity. It is made available under aCC-BY 4.0 International license.

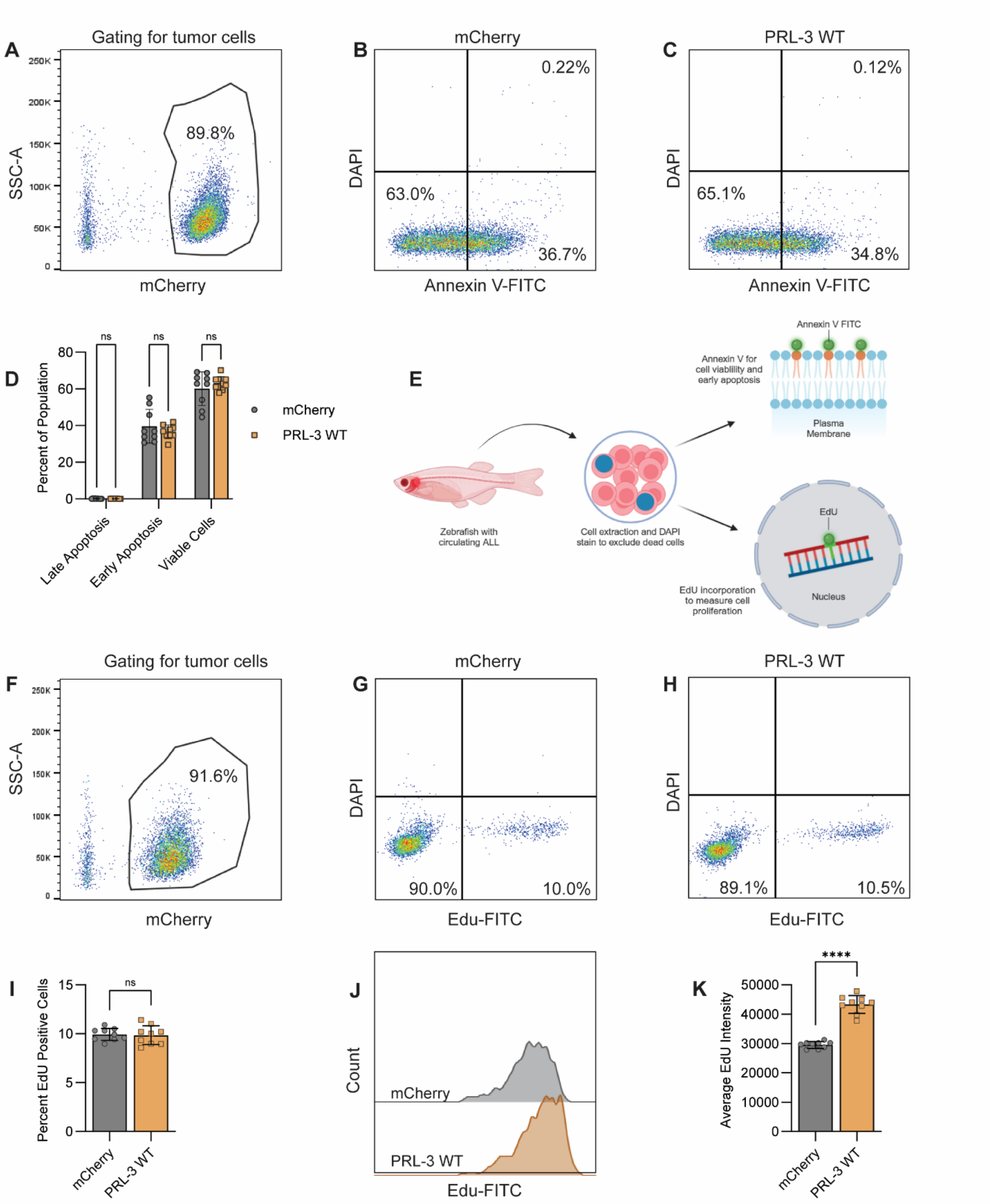

Supplemental Figure 7. Annexin V and EdU analysis of ALL samples.
(A) Representative plot for gating of mCherry-positive tumor cells for Annexin V staining. **(B, C)** Representative Annexin V staining plots for mCherry control and PRL-3 WT groups **(D)** Quantification of Annexin V-positive cells, data points represent three animals per condition. **(E)** Schematic illustrating the methodology for Annexin V and EdU assays. **(F)** Representative plot for gating of mCherry-positive tumor cells for EdU analysis. **(G, H)** Representative EdU incorporation plots for mCherry control and PRL-3 WT groups. **(I)** Quantification of EdU-positive cells. Data points are the pooled reads from three animals per group. **(J)** Representative EdU signal intensity plots for mCherry and PRL-3 WT groups. **(K)** Quantification of the EdU signal intensity per condition. Data points are the pooled reads from three animals per group. Error bars represent standard deviation; significance was determined using an unpaired *t*-test. ****, $p<0.0001$.

bioRxiv preprint doi: https://doi.org/10.1101/2025.05.14.654016; this version posted May 18, 2025. The copyright holder for this preprint (which was not certified by peer review) is the author/funder, who has granted bioRxiv a license to display the preprint in perpetuity. It is made available under aCC-BY 4.0 International license.

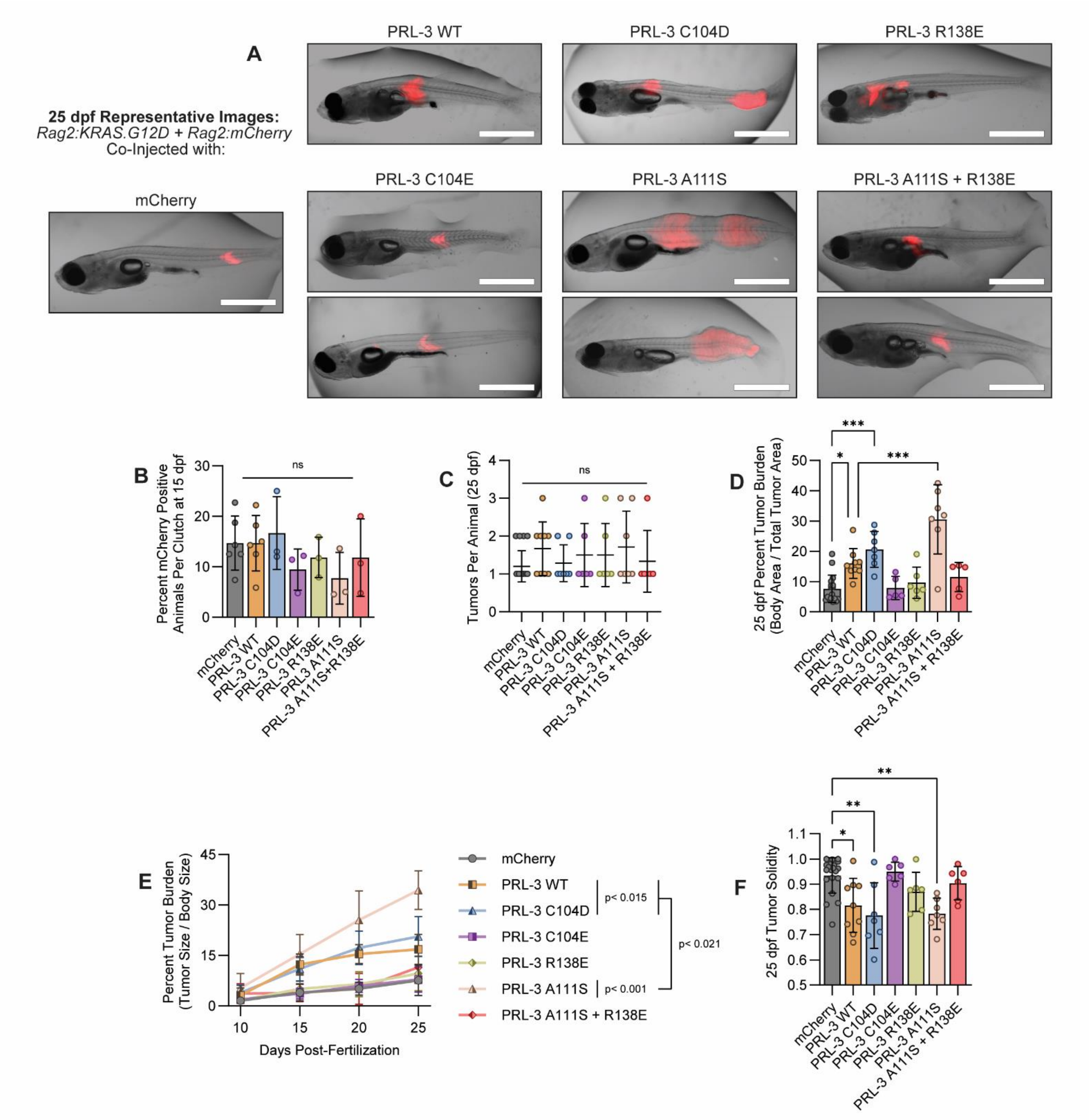

Supplemental Figure 8. Enhancing the phosphatase activity of PRL-3 boosts tumor size in the RMS model in a CNNM-binding-dependent manner.

(A) Representative images of animals injected with the indicated RMS constructs and PRL-3 mutants. **(B)** Quantification of animals per clutch showing detectable mCherry signal at 15 days post-fertilization (dpf); each data point represents one clutch (≥25 animals). **(C)** Quantification of the number of tumors per animal at 25 dpf. Animals with tumor burdens exceeding 50% of their total body size were excluded from analysis. **(D)** Quantification of tumor burden relative to animal body size at 25 dpf. Only the largest tumor was measured in animals with multiple tumors. **(E)** Quantification of tumor burden relative to body size over time. **(F)** Quantification of tumor solidity at 25 dpf. Tumors not located along the animal's torso or tail (such as those on the dorsal fin or jaw) were excluded from the analysis due to differing morphology. Data in C-F were pooled from at least 3 clutches per group; each data point represents an individual animal. Error bars represent standard deviation. Statistical significance was assessed with an ordinary one-way ANOVA with Tukey correction (A, C, E-G), unpaired t-test (B), or two-way ANOVA (D). *, p<0.05; **, p<0.01; ***, p<0.001; ****, p<0.0001.

bioRxiv preprint doi: https://doi.org/10.1101/2025.05.14.654016; this version posted May 18, 2025. The copyright holder for this preprint (which was not certified by peer review) is the author/funder, who has granted bioRxiv a license to display the preprint in perpetuity. It is made available under aCC-BY 4.0 International license.

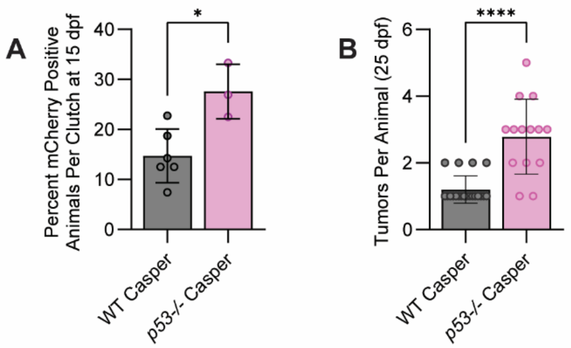

Supplemental Figure 9. Knockout of p53 increases tumorigenesis in the RMS model.
(A) Quantification of animals per clutch with detectable mCherry signal at 15 dpf, indicating tumor presence. Each data point represents an individual clutch of at least 25 animals. **(B)** Quantification of tumor number per animal at 25 dpf. Error bars represent standard deviation. Statistical significance was determined using an unpaired *t*-test. *, $p<0.05$; ****, $p<0.0001$.

bioRxiv preprint doi: https://doi.org/10.1101/2025.05.14.654016; this version posted May 18, 2025. The copyright holder for this preprint (which was not certified by peer review) is the author/funder, who has granted bioRxiv a license to display the preprint in perpetuity. It is made available under aCC-BY 4.0 International license.

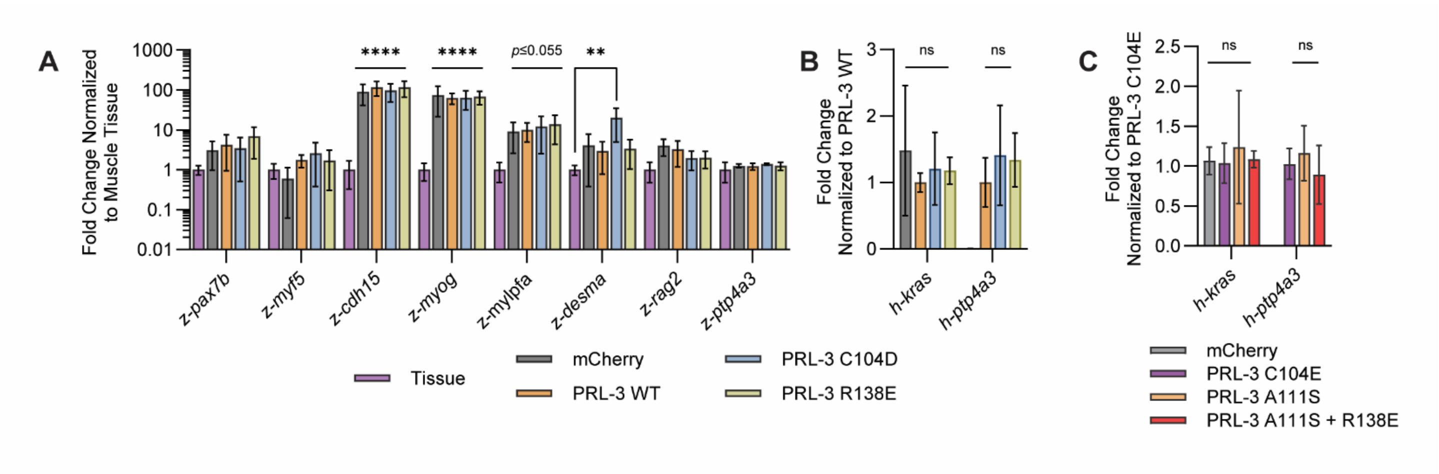

Supplemental Figure 10. The overexpression of PRL-3 mutants has a minimal effect on RMS gene signatures. RT-qPCR results for endogenous (**A**) and injected transgenes (**B**) of interest in RMS samples, with expression normalized to non-cancerous muscle tissue. Each analysis was conducted with samples extracted from six animals per condition. (**C**) RT-qPCR analysis for injected transgenes, normalized to the PRL-3 C104E group, with three animals analyzed per condition. Error bars represent standard deviation. Statistical significance was determined by ordinary one-way ANOVA for each gene of interest, significance is defined as **, p<0.01; ****, p<0.0001

bioRxiv preprint doi: https://doi.org/10.1101/2025.05.14.654016; this version posted May 18, 2025. The copyright holder for this preprint (which was not certified by peer review) is the author/funder, who has granted bioRxiv a license to display the preprint in perpetuity. It is made available under aCC-BY 4.0 International license.

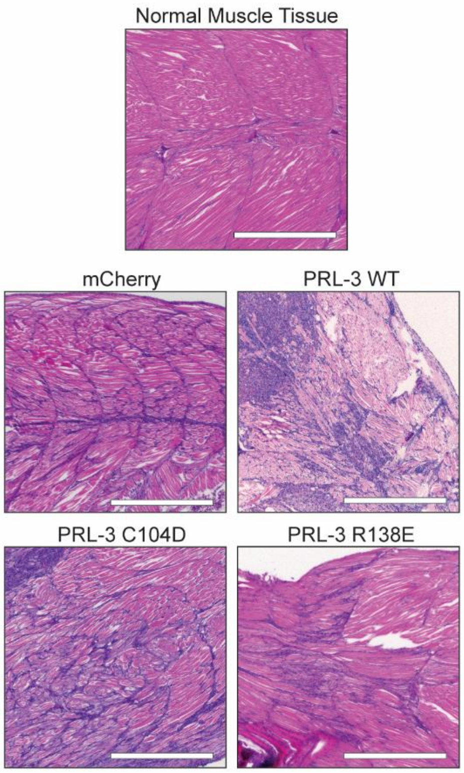

Supplemental Figure 11. Representative H&E images of RMS tumor sections.
Hematoxylin and eosin (H&E) staining of RMS tumor sections from zebrafish injected with rag2:KRAS(G12D) and the indicated PRL-3 transgene, shown alongside normal muscle tissue for comparison. Hematoxylin stains nuclei, while eosin stains cytoplasm and extracellular structures. Scale bar, 500 μm.

bioRxiv preprint doi: https://doi.org/10.1101/2025.05.14.654016; this version posted May 18, 2025. The copyright holder for this preprint (which was not certified by peer review) is the author/funder, who has granted bioRxiv a license to display the preprint in perpetuity. It is made available under aCC-BY 4.0 International license.

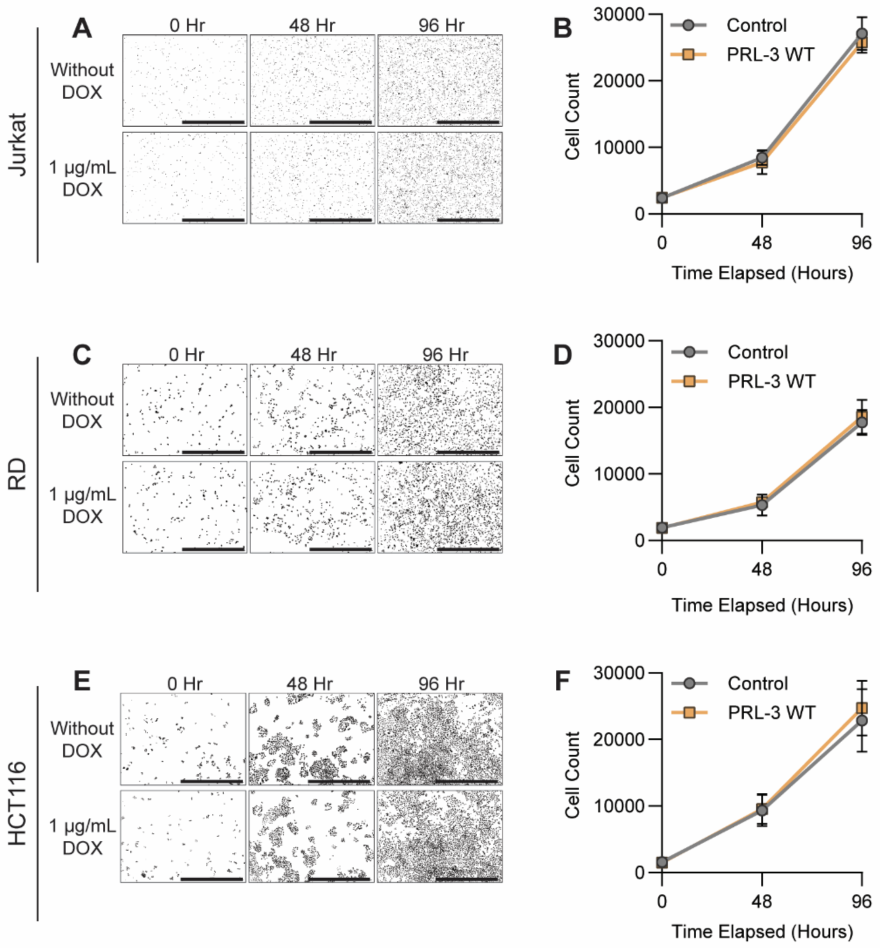

Supplemental Figure 12. The overexpression of PRL-3 does not enhance proliferation in vitro. Representative images showing nuclei counts used to assess proliferation rates in Jurkat (**A**), RD (**C**), and HCT116 (**E**) cell lines with or without PRL-3 overexpression. **(B, D, F)** Quantification of cell numbers over time in the same cell lines under the same conditions. Data represent pooled results from at least three independent experiments for each cell line. Error bars indicate standard deviation. No statistically significant differences were observed between conditions, as determined by *t*-test.

bioRxiv preprint doi: https://doi.org/10.1101/2025.05.14.654016; this version posted May 18, 2025. The copyright holder for this preprint (which was not certified by peer review) is the author/funder, who has granted bioRxiv a license to display the preprint in perpetuity. It is made available under aCC-BY 4.0 International license.

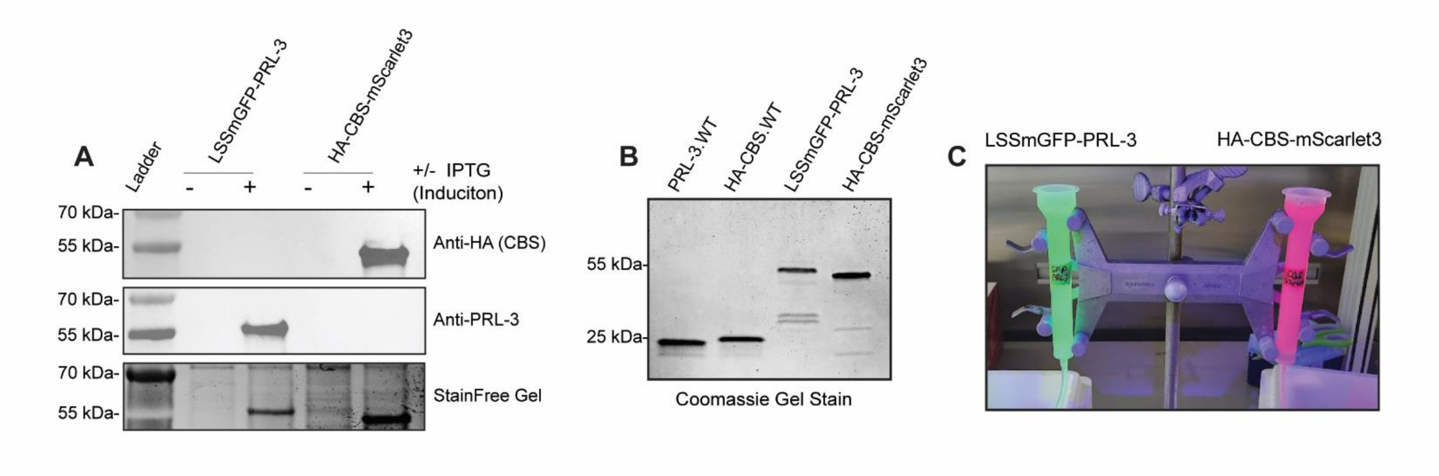

Supplemental Figure 13. Purification of PRL:CBS FRET proteins
(A) Western blot showing recombinant protein expression following IPTG induction. **(B)** SDS-PAGE and Coomassie staining of purified proteins to confirm purity. **(C)** Fluorescence verification of FRET constructs (LSSmGFP-PRL-3 and HA-CBS-mScarlet) during the purification process.

bioRxiv preprint doi: https://doi.org/10.1101/2025.05.14.654016; this version posted May 18, 2025. The copyright holder for this preprint (which was not certified by peer review) is the author/funder, who has granted bioRxiv a license to display the preprint in perpetuity. It is made available under aCC-BY 4.0 International license.

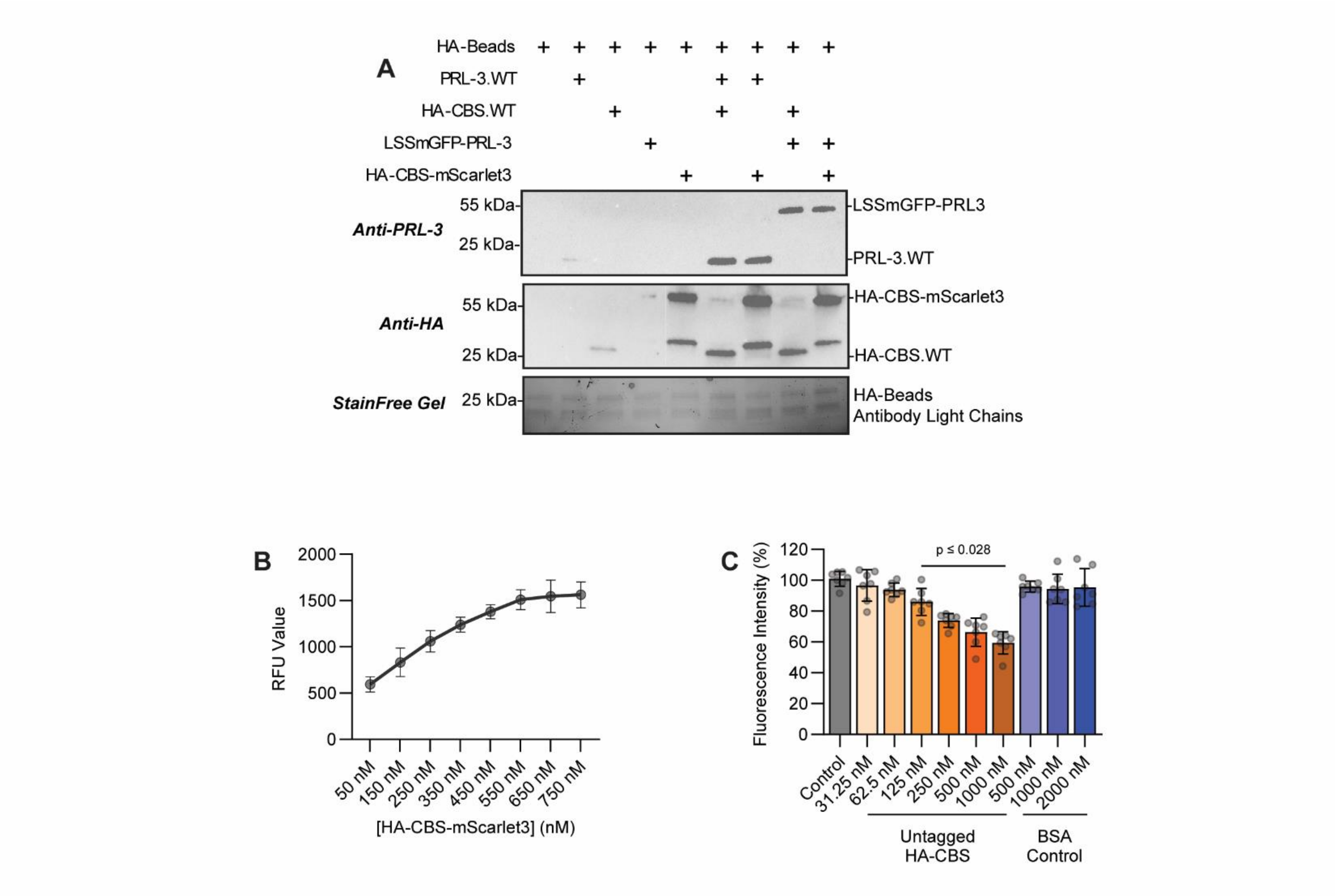

Supplemental Figure 14. Validation of the PRL:CBS FRET pair.

(A) In vitro immunoprecipitation assay using the FRET fusion proteins compared to their non-fluorescent protein counterparts. **(B)** FRET signal saturation observed upon addition of increasing concentrations of HA-CBS-mScarlet to a constant 250 nM concentration of LSSmGFP-PRL3. **(C)** Competitive FRET assay showing reduced signal with increasing concentrations of HA-CBS designed to compete for LSSmGFP-PRL3 binding and displacing HA-CBS-mScarlet. BSA was used as a negative control. Data are representative of two independent experiments, normalized to untreated controls. Error bars indicate standard deviation. Statistical significance was determined with ordinary one-way ANOVA and Tukey correction and is indicated in panel C.

bioRxiv preprint doi: https://doi.org/10.1101/2025.05.14.654016; this version posted May 18, 2025. The copyright holder for this preprint (which was not certified by peer review) is the author/funder, who has granted bioRxiv a license to display the preprint in perpetuity. It is made available under aCC-BY 4.0 International license.

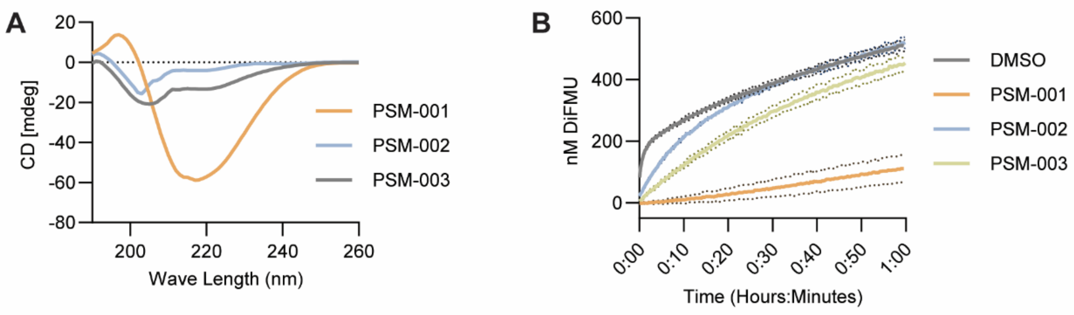

Supplemental Figure 15. Peptide circularization strategies produce PRL-substrate mimics (PSMs) with variable structural and functional properties.
(A) Circular dichroism (CD) spectra of each PSM peptide, illustrating differences in secondary structure. **(B)** DiFMUP phosphatase assay results assessing the inhibitory efficacy of each peptide against PRL enzymatic activity. The data shown are representative of at least two independent experiments. Dotted lines (B) indicate standard deviation.

bioRxiv preprint doi: https://doi.org/10.1101/2025.05.14.654016; this version posted May 18, 2025. The copyright holder for this preprint (which was not certified by peer review) is the author/funder, who has granted bioRxiv a license to display the preprint in perpetuity. It is made available under aCC-BY 4.0 International license.

Supplemental Table 1: *Site-Directed Mutagenesis Primers*

Mutation:	**Forward (Sense):**	**Reverse (Anti-Sense):**
PRL-3 C104D	CAGGCCCGCCACGTCGTGCACAGCCACG	CGTGGCTGTGCACGACGTGGCGGGCCTG
PRL-3 C104E	CCCAGGCCCGCCACCTCGTGCACAGCCACGC	GCGTGGCTGTGCACGAGGTGGCGGGCCTGGG
PRL-3 A111S	CACAAGGACTGGACTCCGGCCCAGGCCC	GGGCCTGGGCCGGAGTCCAGTCCTTGTG
PRL-3 R138E	GCTGTTGATGGCTCCCTCGCGCTTCTGGCGGAT	ATCCGCCAGAAGCGCGAGGGAGCCATCAACAGC
PRL-3 C170S	CTACATAACGCAGCTCCGGGTCTTGTGCG	CGCACAAGACCCGGAGCTGCGTTATGTAG

Plasmid Name	Additional Identifying Information	Purpose	Sequence
pENTR-PRL3-WT	Sourced from pENTR-D-TOPO	Entry vector containing human PRL-3 WT	CAACGCGGCCT
pME-mCherry	Middle Entry Vector from Tol2 Kit	Entry vector containing mCherry	CTTTCCTGCGT
pLPC-N Flag	Addgene #12521	Lentivirus destination vector with 3x Flag tag on N-terminus	TAGAAAAGATC
pLenti CMV Puro DEST	Addgene #17452	Lentivirus destination vector	GTTCTTTCCTGCG
pCW57.1	Addgene #41393	All-in-On doxycycline inducable lentivirus gateway plasmid	AATTCTCGACCTC
psPAX2	Addgene #12260	Lentivirus packaging plasmid	TTGATTATTGACTA
pCMV-VSV-G	Addgene #8454	Lentivirus packaging plasmid	GAGGCGGTTTGC
Rag2.GW.DEST	zRag2.Gateway.DEST	Destination plasmid with zebrafish Rag2 promoter	AGCGCCCAATAC
Rag2.mMyc	zRag2.GW.mMyc. Rag2 mouse myc	Zebrafish Rag2 promoter with mouse Myc	AGCGCCCAATAC
Rag2.hKRAS.G12D	zRag2.GW.KRAS.G12D, Rag2 human k-ras G12D mutant	Zebrafish Rag2 promoter with human KRAS.G12D	AGCGCCCAATAC
pET.28b(+).LSSmGFP.PRL3.C104D	GFP.PRL3 FRET Pair 1	GFP-PRL3 FRET protein expresstion vector	TGCTGGTTGCCA
pET.28b(+).HA.CBS.mScarlet3	HA.CBS.Scarlet FRET Pair 2	HA-CBS-mScarlet FRET protein expression vector	TGCTGGTTGCCA

bioRxiv preprint doi: https://doi.org/10.1101/2025.05.14.654016; this version posted May 18, 2025. The copyright holder for this preprint (which was not certified by peer review) is the author/funder, who has granted bioRxiv a license to display the preprint in perpetuity. It is made available under aCC-BY 4.0 International license.

Supplemental Table 3: *RT-qPCR Primers*

Target Gene:	**Forward (Sense):**	**Reverse (Anti-Sense):**	**Purpose:**
m-cMyc	AGCGACTCTGAAGAAGAGCAA	GCACCTCTTGAGGACCAGTG	Transgene Control
h-KRAS.G12D	TTGTGGTAGTTGGAGCTGATG	GACCTGCTGTGTCGAGAATATC	Transgene Control
h-PTP4A3	GTGTGTGTGAAGTGACCTATGA	CTCACAGAACTTGGCCTTCA	Transgene Control
z-eef1a	CTACCCTCCTCTTGGTCGCT	ACGGTGTGATTGAGGGAAATTCA	House Keeping
z-rplp0	GCGTCCCTACCGTGAGATTTT	TTGGGGTAGTCATCCAGCAGT	House Keeping
z-rag2	AGCTCTCAGATTTCGGAGTACAC	ACAAGGCTGCCACAATTCAC	Lymphocyte Marker
z-rag1	AGCAATGATGCAAGGCAGAG	TGTGCAGGGGCTGGAATATC	Lymphocyte Marker
z-lck	AGAAGATCTCGATGGTTTGTCTGT	CGCAGTTCCCCATGTTTACG	T-cell Marker
z-tcr β-c2	ATTCACCTGCACTGTCCGAT	AGCTTCAATCCCTTCGGCTT	T-cell Marker
z-pax5	CTGATTACAAACGCCAAAAC	CTAAATTATGCGCAGAAACG	B-cell Marker
z-igD-VH1	GAGAGCAGCAAAAGGATGGC	TGCAAGTTTGGTCTTGTTCTGC	B-cell Marker
z-IgM-VH1	CATGACAATGGATATTGTGTCC	ACATGAAGGTTGCTGATCCAC	B-cell Marker
z-pax7b	GGAAGTAAACCTAGACAGGTGGC	GGCCTCACCTGAGGGAACTG	ARMS Marker
z-myf5	GCTGCTCAGAGAGCATGGTTG	TCCACGATGCTGGACAAACAC	Muscle Progenitor Marker
z-cdh15	GCCGCTCTGAGTTACTCCAT	CTGAAGGACTGCACCACATCT	Myoblasts Marker
z-myog	GCTCCACATACTGGGGTGTC	GAGTCGTCGTTCAGCAGATCC	Muscle development Marker
z-desma	CTCAGTGCTCAGCGTGTCG	CGGTAATGGGTCACGGCAAT	Intermediate filament Marker
z-mylpfa	CGGAGAGAAGTTGAAGGGTGC	ATCTCCTCTGCGGTGAACCT	Muscle marker

bioRxiv preprint doi: https://doi.org/10.1101/2025.05.14.654016; this version posted May 18, 2025. The copyright holder for this preprint (which was not certified by peer review) is the author/funder, who has granted bioRxiv a license to display the preprint in perpetuity. It is made available under aCC-BY 4.0 International license.

Supplemental Table 4: *Cyclic Peptide Sequences and Linkers*

**PSM-001**	GVQKVNNEGEGDPFYEVLN, Head-to-tail amide cyclic
**PSM-002**	GVQKVNNEGEGDPFYEVL-(D-Pro), Head-to-tail amide cyclic
**PSM-003**	PVQKVNNEGEGDPFYEVL-(D-Pro), Head-to-tail amide cyclic
